# Environmental and Health Effects of Ventilation in Subway Stations: A Literature Review

**DOI:** 10.3390/ijerph17031084

**Published:** 2020-02-08

**Authors:** Yueming Wen, Jiawei Leng, Xiaobing Shen, Gang Han, Lijun Sun, Fei Yu

**Affiliations:** 1 School of Architecture, Future Underground Space Institute, Southeast University, Nanjing 210019, Jiangsu, China; wenyueming66@163.com (Y.W.); han_gang007@163.com (G.H.); s-lijun@163.com (L.S.); 230189387@seu.edu.cn (F.Y.); 2 School of Public Health, Station and Train Health Institute, Key Laboratory of Environmental Medicine Engineering, Ministry of Education, Southeast University, Nanjing 210019, Jiangsu, China; 101006194@seu.edu.cn

**Keywords:** subway, ventilation, environmental effect, health effect, mitigation measure, review

## Abstract

Environmental health in subway stations, a typical type of urban underground space, is becoming increasingly important. Ventilation is the principal measure for optimizing the complex physical environment in a subway station. This paper narratively reviews the environmental and health effects of subway ventilation and discusses the relevant engineering, environmental, and medical aspects in combination. Ventilation exerts a notable dual effect on environmental health in a subway station. On the one hand, ventilation controls temperature, humidity, and indoor air quality to ensure human comfort and health. On the other hand, ventilation also carries the potential risks of spreading air pollutants or fire smoke through the complex wind environment as well as produces continuous noise. Assessment and management of health risks associated with subway ventilation is essential to attain a healthy subway environment. This, however, requires exposure, threshold data, and thereby necessitates more research into long-term effects, and toxicity as well as epidemiological studies. Additionally, more research is needed to further examine the design and maintenance of ventilation systems. An understanding of the pathogenic mechanisms and aerodynamic characteristics of various pollutants can help formulate ventilation strategies to reduce pollutant concentrations. Moreover, current comprehensive underground space development affords a possibility for creating flexible spaces that optimize ventilation efficiency, acoustic comfort, and space perception.

## 1. Introduction

Subways, a typical type of urban underground space (UUS), are the most commonly used mode of public transportation, and are gradually becoming an indispensable component of large cities. With rapid urban development, transit-oriented developments (TODs) and are gradually becoming a new urban model [1,2,3,4]. Subways, dubbed “the lifeline of urban development,” connect city regions, relieve road traffic congestion, and provide hubs of interest and commerce in the underground network [5,6,7]. Compared with cars and buses, the subway is a low-carbon transport mode and is crucial in meeting climate goals. Today, huge populations of passengers and limited subway facilities are causing deterioration in indoor subway environments, particularly during rush hours. In China, daily passenger volumes of Beijing and Shanghai subways are more than 10 million. As a consequence, confined and crowded subway environments carry potential health risks and may trigger acute and chronic health issues as well as transmit epidemic diseases [8,9,10,11,12]. 

Safety and environmental health in subways has become increasingly important and attracted attention from numerous researchers in recent decades. However, the lack of long-term-effect research and limited detection technology may lead to the neglect of the environmental health effects of subways. Health effects and pathogenesis are complex due to interactions of risk factors, such as additive, synergistic, or cumulative reactions [13]. Therefore, comprehensive reviews and summaries are necessary for further research and overall environmental optimization. To date, five representative reviews [14,15,16,17,18] on the health effects of subways or other types of UUS show the main content of the existing research. These reviews show that there is ongoing research into environmental health in UUS, mainly in areas such as the physical environment, mental health, and human behavior. The majority of the research centers on the physical environment, particularly indoor air quality (IAQ). However, these reviews focus only on single areas (toxicology, pathology, epidemiology, environment, or engineering) and rarely examine these areas in combination and offer practical mitigation measures.

A subway space is usually isolated from the atmospheric environment, and its hermetic nature may lead to heat and moisture accumulation. Various sources of pollution can result in the accumulation, breeding, and transmission of harmful substances. Additionally, complex ambient airflows referred to as “subway climate”, affects the dispersion of air pollutants, fire smoke, and biological or chemical agents [19,20,21]. Therefore, ventilation significantly affects the health and safety of subway passengers and workers [10,22]. This paper connects relevant studies from engineering, environmental, and medical aspects and discusses the environmental and health effects associated with subway ventilation systems. This paper is of certain significance to future theoretical research and practical application.

## 2. Methodology

In this paper, we have attempted to answer three crucial questions:

1. What environmental factors are affected by ventilation systems in subway stations?
2. How do these environmental factors affect human health?
3. How can ventilation improve environmental health in subway stations?

Because of the wide range of environmental and health effects, it was not feasible to conduct a single systematic review. This narrative review used the following search terms “underground space”, “subsurface space”, “subway”, “metro”, “tube”, “underground railway”, together with “health”, “well-being”, “health impact”, “health effect”, “health risk”, “health implication”, “exposure assessment”, and as well as “ventilation”, “filtration”, “purification”, “wind environment”, “airflow”, “air quality”, “sick building syndrome (SBS)”, and “building-related illness (BRI)”. The search literature databases include PubMed, Medline (Ovid), Science Direct, Scopus, Google Scholar, and through websites including the Associated Research Centers for the Urban Underground Space (ACUUS), the World Health Organization (WHO), and various government websites including construction, environment, and health ministries since 1980 to 2019. As this was not a systematic but narrative review, no formal validity/quality, risk of bias assessments were done, and no exclusion criteria were applied. Through literature retrieval and analysis, the review framework expanded to other effects not previously considered, like “fire evacuation” and “noise.” 

## 3. Results

There has been a limited review of the health effects due to poor ventilation for both aboveground or underground buildings. Seppänen and Fisk reviewed human responses to ventilation on aboveground buildings but mainly focused on epidemiological statistics of responses and diseases [23]. Sundell et al reviewed the correlation between ventilation rates and SBS symptoms in offices [24]. More importantly, the related WHO guidelines and “The WELL building standard” briefly state some health risks and control measures [25,26,27,28,29,30,31]. According to the results of the literature search, as shown in Table 1, Table 2, Table 3 and Table 4, ventilation directly affects human health and further, indirectly through environmental factors. Firstly, ventilation can ensure a comfortable thermal environment and inhibit the growth of microbes. Secondly, ventilation is indispensable to improve IAQ in subway stations. Thirdly, the complex wind environment significantly affects the draft sensation and pollutant transmission. Lastly, ventilation devices expose passengers and workers to background noise for the long term. As a result, ventilation not only acts as a mitigation measure but also a source of exposure. 

**Figure 1:**
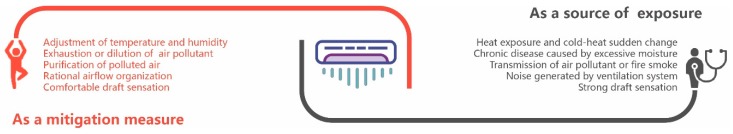
Dual effect of subway ventilation.

**Figure 2:**
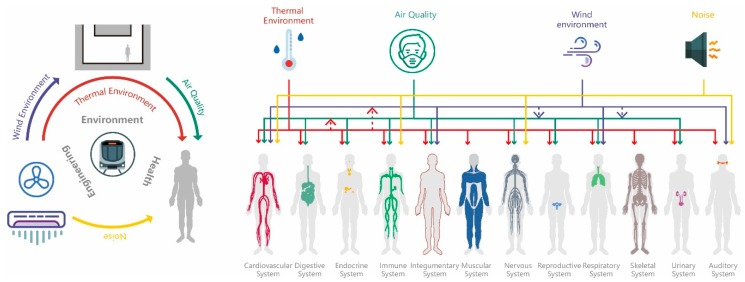
Mechanism of subway ventilation effects on environmental health.

The structure of this paper is based on the above aspects and results. In Section 4 “Ventilation as a mitigation measure” and Section 5 “Ventilation as a source of exposure”, the dual effect (Figure 1) and mechanism (Figure 2) are analyzed from the environmental effects and corresponding health effects. Meanwhile, some mitigation measures are proposed. In Section 6 “Discussion”, summaries, questions, deficiencies, and hypotheses are discussed from three levels, from problem characteristics to assessment basis to mitigation measures.

## 4. Ventilation as a Mitigation Measure

Due to the limited connection with the atmospheric environment, natural ventilation is not only unable to meet the strict subway ventilation requirements but also disrupts indoor airflow. Mechanical ventilation is a reliable measure for optimizing the thermal environment and IAQ. A subway ventilation system is composed of multiple systems that cooperate and coordinate with each other, including heating, ventilating, and air conditioning (HVAC) systems and platform screen doors (PSDs). Intelligent ventilation systems are capable of adjusting their mode of operation based on external and internal conditions and thereby ensure a comfortable, healthy, thermal environment and IAQ in subway stations. The failure of a subway ventilation system to adjust the physical environment in a timely and effective manner will incur a number of health effects, which will be discussed in the following sections.

### 4.1. Thermal Environment

The thermal environment in subways significantly affects human comfort and health, as well as related environmental factors such as IAQ, airflow, and sound transmission. The water vapor condensation, humidity radiation, and indoor air pollution lead to high temperature and humidity in a subway environment. Exposure to heat and cold has extensive effects on almost every body system, including the cardiovascular, digestive, endocrine, immune, skin, muscular, nervous, reproductive, respiratory, skeletal, urinary, and auditory systems [25,31,32].

#### 4.1.1. Temperature

Any impairment of thermoregulation may increase the risks of heat-related diseases [33]. The thermal environment in a subway is influenced by periodic outside climate, complex station structure, variable facility operations, and fluctuating passenger numbers. Subway gathers a considerable number of passengers within a closed space. Despite being used for a short time, uncomfortable temperature and humidity and poor air quality have various health effects [34,35,36]. Exposure to excessive heat may confuse cardiovascular, endocrine, immune, integumentary, urinary, and nervous systems and then cause physiological responses (heat stroke, syncope, cramp, dehydration, and then multiple organ dysfunctions) and heart diseases [37]. Additionally, high ambient temperature can aggravate the harm of noise on the auditory system [38] and abate digestive function [32]. Heat-related mental symptoms like anxiety and dysphoria also have serious potential risks [39]. 

On the contrary, a subway environment is not too cold, because it has good thermal stability and various heat sources. Nevertheless, improper ventilation may cause a cold environment or alternate heat and cold. The human body is more sensitive to cold stimuli than to warm [40]. On the one hand, sudden cold exposure can induce acute ischemic heart disease (IHD) and affects more profoundly than chronic IHD on acute myocardial infarction [41]. The physiological responses caused by cold exposure include elevated blood pressure, dehydration, atherosclerosis, and myocardial injury [42]. On the other hand, cold, dry, and windy environments can cause rhinitis, obstructive airway, and other respiratory responses [43]. Cold exposure may cause vasoconstriction in respiratory tract mucosa and suppress immune responses and barrier functions, which are responsible for increased susceptibility to infections [44,45,46].

#### 4.1.2. Humidity

Humidity directly affects passengers’ environmental perception and exerts potential risks to human health. On the one hand, high humidity can cause discomfort, especially when the relative humidity (RH) is above 70% [47,48]. Significant headaches were observed when CO_2_ concentrations increased to 12,000 ppm at an RH of 85% [49]. Furthermore, high humidity is closely associated with the perception of odor, “stuffy air,” skin, and upper airway symptoms [50,51,52,53,54]. Prolonged exposure to high temperature and humidity may lead to functional attenuation of reproductive, muscle, and skeletal systems [55,56]. On the other hand, humidity affects the formation and spread of air pollutants such as particles, aerosols, and microbes. But the underlying mechanisms among humidity, air pollutant, and human health need further studies [57,58,59,60,61]. High humidity may promote the accumulation and growth of microbial pathogens [62] and increase off-gassing, for example, an increase in RH of 35% can increase the missions of formaldehyde by a factor of 1.8–2.6 [25]. Because of the limitation of ventilation, underground space usually has sufficient moisture, which provides an available condition for the growth of bacteria and fungi, in particular, filamentous fungi (mold). Therefore, preventing persistent dampness can minimize microbial pollution in subways [62,63].

On the contrary, low humidity affects perceived air quality (PAQ), sensory irritations, work performance, and virus survival [58]. The perception of dry air is most likely related to sensory irritants in desiccated mucous membranes or eyes caused by insufficient ventilation resulting in air pollutants [64,65]. Firstly, some ophthalmologic studies showed that low relative humidity (RH) led to break-up or thinning of the eye tear film resulting in less tear production or exacerbation of water loss, which causes desiccation and hyperosmolarity in the eye tear film and initiation of a cascade of inflammatory reactions [58,66]. Secondly, both low and high RH can alter the mucous viscosity and the mucociliary activity in nasal regions, which affects bacterial adherence and penetration of foreign species. Low RH may cause desiccation or dehydration of nasal epithelium and, therefore, worsen the inflammations [67,68]. Thirdly, cold and low RH conditions are conducive to the survival and transmission of some influenza viruses like respiratory syncytial virus, human rhinovirus, and avian influenza virus [57,69,70], but the opposite has been observed for dust-mite allergens and other virus types [57,71,72].

#### 4.1.3. Mitigation Measures

HVAC systems can effectively adjust the thermal environment of subway stations. However, identifying environmental indices that ensure human health remains a challenge. Evaluation standards for thermal environment include three categories, of survival, comfort, and efficiency. There are several evaluation indices such as teq (temperature equivalent), ET (effective temperature), ET*, SET* (standard effective temperature), HIS (heat stress index), WBGT (wet bulb globe temperature index), and PMV (predicted mean vote). The PMV-PPD (predicted percentage dissatisfied) evaluation system, included in ISO (International Organization for Standardization) 7730 [73], defines a thermal environment as a function of four physical variables (air temperature, mean radiant temperature, relative air velocity, and air humidity) and two anthropogenic variables (activity level and clothing) [74]. In the popularization of the PMV-PPD system, some problems and inefficiency appear, such as climate, altitude, population density, living habits, indoor activity, sweat evaporation, and psychological expectations. Therefore, adaptive models and correction factors have been proposed to improve the suitability and accuracy of the system [75,76]. Thermal indices of subways should keep higher standards and change timely to cope with the changing environment, because complex airflows and crowded population may quickly deteriorate indoor environments and affect human comfort and health.

Rational designs of entrances and shafts can prevent outdoor rain, moisture, and cold air from entering subway stations. To be more specific, first, entrances and shafts should be kept away from underground water, streets, and close to urban air corridors and wind directions, so that air pollutants or heat can be removed quickly from aboveground surroundings. Next, roof, barricade, air curtain, and green wall can prevent rainwater and cold wind from entering the station. Then, increasing length, section, and area of entrance channel, and installing an HVAC system obviously buffer inside and outside temperature differences. Finally, plane shapes of entrance channels, such as linear, broken linear, or L-shaped, can be designed according to the demand of using piston wind. Additionally, controlling water seepage and accumulation in tunnels reduces the sources of humidity and air pollutants. Similarly, controlling the water sourced from station toilets, cleaning rooms, and equipment improves the thermal conditions and IAQ. Furthermore, the condensate water of the air-conditioning system is still a problem, which produces mold and other microbes. Conventional air-conditioning systems share a cold source and simultaneously cools and dehumidifies the air to remove heat and moisture. Because of the temperature difference between cooling (below the air) and dehumidification (below the dew point), conventional systems increase energy waste and condensate water, because they can only accurately control one parameter of temperature or humidity and hence, cause an uncomfortable sensation of “too cold” or “stuffy”. Temperature and humidity independent control systems can address this problem and adjust flexibly for different subway zones [77,78]. In this independent control system, the liquid desiccant system engages all fresh air load and indoor wet load and adjusts the air supply humidity. Meanwhile, the high-temperature chiller engages most of the indoor sensible heat load and adjusts the air supply temperature. Additionally, calculation and prediction of heat and humidity loads need further studies due to the variations of cold air infiltration, passenger flow, and heat source in subways.

### 4.2. IAQ

Confined subway spaces tend to accumulate air pollutants generated from indoor or outdoor [79,80,81,82]. Although passengers usually use subways for about one hour a day (20 minutes in stations), it is enough to expose passengers to an unhealthy environment and the environmental hygiene in the carriages is usually worse than in the stations. In Barcelona, commuters spend about 3% of the day in the subway, but this microenvironment may account for up to 47% of the total particulate matter (PM)2.5 daily dose [83]. Besides, lots of inhalable particulate matters generate inside subways and thus have differences from PMs entering from outside [80,84,85,86,87]. Hence, it is necessary to specialize in the health effects of subway air. Several representative reviews summarized the research process and primary objects of IAQ in subways [16,18,80,86]. Based on these reviews, particulate matters (PMs), volatile organic compounds (VOCs), and bioaerosols are three main factors affecting IAQ. Also, other excessive or deficient air components can affect human health such as CO, CO_2_, NO_2_, and O_3_. Instead of establishing a logic for health risk control, these reviews focused on certain aspects of health effects. This paper clarifies a path of air pollutants from generation to impact to reduction. Sources and compositions are the basis of controlling pollutant generations and studying their toxicities. Although relevant studies aboveground have certain reference significances, studies specialized in subway pollutants are more conducive to formulate environmental indices for risk control.

#### 4.2.1. PMs

PMs are the most concerned research object of air pollutants in subways. Subway PMs’ sources are from both outdoor and indoor: (1) aboveground atmosphere and surrounding environment, and (2) underground tunnels, stations, facilities, and passengers. The compositions and characteristics of subway PMs are mainly determined by indoor sources, including mechanical wear, material degradation, tunnel dust, and human activities like cleaning. Wheel and brake pads, steel rails, and power supply materials give the particles a peculiarly metalliferous character. Subway PM concentrations are directly affected by frequency of train, commuter influx, ventilation system, surrounding air, soil resuspension, construction year, station depth, and other pollutant concentrations [11,80,86,87,88,89,90,91,92,93,94,95,96,97,98,99,100,101,102,103,104,105,106,107,108,109]. Because of the complexity of the indoor and outdoor environments, there is no consistent composition of subway PMs. The main components are metal, total carbon (TC), and secondary inorganic aerosol (SIA) [97,108,110,111,112,113]. Firstly, subway PMs are highly ferruginous (FePM) and accompanied by trace metals, such as Mn, Cr, Cu, Sb, Ba, Zn, and Mo, of which the dominant ferruginous component is typically oxidized to magnetite, maghemite, and hematite [88,90,94,96,102,103,106,110,114,115,116,117,118,119,120,121,122,123,124,125,126,127,128,129,130,131,132,133,134,135,136,137]. Secondly, carbonaceous PM is the first or second most abundant component in subways, especially platforms [110,138,139,140,141]. Thirdly, the crustal particles (mainly silicates) are not only from the mineral dust in infiltrated outdoor air but also from construction material and rock soil inside subways. Halite and secondary inorganic compounds like water-soluble nitrate, sulfate, and ammonium are rare in subway air and usually infiltrate from outside [80,98,110,111,121,137].

PMs of different sizes can reach different human organs and exert specific health effects [142]. Smaller PMs penetrate deeper parts of the lungs and do more harm. For the people with the same susceptibility and exposure time, exposure doses are mainly dependent on particle sizes and concentrations [83]. Furthermore, PMs may combine with other air pollutants and generate secondary pollutants, such as nitrogen oxides [143]. PMs’ concentration presents spatiotemporal characteristics in subways. Urban environments, meteorological variations, subway conditions, applied technologies, route choices, and personal habits all lead to variable exposure levels to air pollutants. Therefore, it is difficult to conclude a meaningful global average for conditions above or below ground [80,144]. In general, PM concentrations measured on subway platforms are several times higher than those recorded simultaneously outside [79,83,104,110,115,145,146,147,148]. 

Studies on pathological and toxicological responses of the human body provide a quantitative basis. Nevertheless, research on cell damage and the health effects of specific physical properties or chemical compositions of subway particles are still insufficient [80,149]. Inhaled PMs can cause cellular inflammation, reactive oxygen, and genotoxic effects and induce acute and chronic responses in different body systems such as skin, ocular, respiratory, and cardiovascular systems. These responses may increase the incidences of heart disease, stroke, allergy, asthma, cancer, and infectious diseases [150,151]. Despite a controversy over the hazards of subway air compared with ground air, there is a consensus on the oxidative potential (OP) of subway PMs. However, oxidation mechanisms of subway PMs are controversial. Some earlier studies implicated that ferruginous nature was responsible for the OP of subway PMs. However, Moreno suggested that although Fe dominated the composition of subway PMs, it was not responsible for oxidative damage and that more likely candidates were the trace metals such as Mn, Zn, Ba, and especially Cu [80,86,99,120,152,153,154,155,156,157,158,159,160,161,162,163,164,165,166,167,168].

#### 4.2.2. VOCs

Subway environments expose passengers to a high level of VOCs, particularly during rush-hours. Subways account for 10%–20% of daily VOC exposure [169]. The longer the passengers stayed in subways, the higher their VOC exposure. Existing research mainly includes aromatic hydrocarbons, carbonyls and chlorinated hydrocarbons (CHs), especially polycyclic aromatic hydrocarbons (PAHs), toluene, HCHO (formaldehyde), and acetaldehyde [138,169,170,171,172,173,174,175,176,177]. In this paper, HCHO is discussed in the VOCs section, although this is controversial in different IAQ guidelines.

Subway VOCs have complex sources of indoor emissions and outdoor infiltrations [80,139,169,170,174,177,178,179,180]. PM-bounded VOC is an important transmission form that is convenient for sampling, measurement, and trace tracking. Firstly, research on aromatic hydrocarbons mainly focus on PAHs and BTEX (benzene, toluene, ethylbenzene, xylenes) and indicates that the highest exposure risk is benzene. Traffic-related emission is a dominant source of BTEX through indoor and outdoor air exchange [139,169,174,180]. BTEX also has many indoor sources, like accidentally spilled oil and various solvents [169,174,177]. Concentrations of aromatic hydrocarbons are mainly affected by seasonality, rail maintenance activities, passenger numbers, outdoor air, and ground transports [80,139,174]. Meanwhile, underground fast-food courts, restaurants, and shopping malls may bring VOCs into subways through airflows [174]. Secondly, carbonyls mainly come from the automobile exhaustion or incomplete combustion of fossil fuels [181]. However, subway trains do not use fossil fuel but electric power. As a result, subway carbonyls mainly come from outdoor air and keep to low levels. Indoor sources also include passengers, materials, and solvents. Besides, ozone chemistry can help to generate aldehydes by reactions with VOCs and unsaturated organic chemicals [170,178,179]. HCHO is the most abundant carbonyl, followed by acetaldehyde and acetone. Concentrations of carbonyls correlate with years of carriages and stations, area, and depth of platforms [179,182]. Although the concentrations meet some health standards, there still exist health risks of VOCs when long-term subway users are exposed to a certain time and doses [170,173,174,178,179,183,184,185]. Thirdly, CHs source from indoor and outdoor air exchange or products containing chlorinated solvents. For example, trichloroethylene and para-dichlorobenzene come from cleaning and deodorizing products through vapor intrusion from toilets and underground water [174].

VOCs affect the human body through inhalation, ingestion, and dermal sorption [179,186]. Exposure to VOCs, particularly those classified as known or suspected carcinogens like benzenes, aldehydes, and ketones, potentially exert adverse effects on human health. Besides, VOCs are essential precursors of many secondary pollutants, which in turn deteriorate IAQ [4,170,174,179,187]. Because PAHs have different carcinogenic activities, their concentrations are not appropriate means for health risk assessments. Better means to estimate the carcinogenic potency of the compounds are to multiply their respective concentrations and toxic equivalency factor (TEF) values. Benzo(a)pyrene (B(a)P) and dibenzopyrene isomers are appropriate indicators for TEF to assess total health risk. B(a)P had the highest carcinogenic potency, but some PAHs still lack toxicological data, TEF values, or analytical methods [172,183,188]. In addition to cancer risk, VOCs also have other adverse effects. Some PAHs are genotoxic, such as B(a)P [139]. Formaldehyde may stimulate respiratory, skin, ocular, and nervous systems, increase incidences of asthma or allergy, and cause DNA adduct formation and clastogenic effects [81,189]. Additionally, the long-term effects of subway VOCs need more data and studies [170,176].

#### 4.2.3. Bioaerosols

Bioaerosols refer to aerosols containing biological particles, such as bacteria, fungi, viruses, and parasite eggs, and have infectivity, pathogenicity, and allergenicity. Subways are high-risk places for microbial growth and propagation due to various sources, thermal environment, limited ventilation, and crowded population [190,191,192]. Existing research mainly focuses on concentration level, diversity, sources, and virulence- and survival-associated properties. Worldwide field research shows different microbial components in subways, of which common microbes include aspergillus, alternaria, bacillus, cladosporium, chrysosporium, geotrichum, micrococcus, propionibacterium, penicillium, and staphylococcus [190,191,193,194,195,196,197,198,199].

Sources and compositions of microbes are affected by various factors such as humans, soil, water, outdoor air, location, depth, and building materials [98,190,192,195,196,197,198,200,201,202]. First, according to genera comparisons, subway microbes are closely associated with the human body and activity [190,200,201]. Foods and garbage produced by passengers also pollute indoor microbial environments [203]. Next, continuous and efficient air exchanges maintain healthy microbial concentrations and make microbial species in subways similar to outside air [201]. Especially during off-peak hours, size distributions of subway bioaerosols are similar to that of outside air [204]. Leung et al. compared microbial communities in Hong Kong with those in the United States and founded different microbial clustering based on continental geography [190]. Then, shallow stations highly correlate with the outdoor conditions [205,206]. Deep tunnels provide a specific microenvironment reflecting indoor air, which may significantly influence the fungal species composition [202,207]. Finally, common genera from the underground mycobiota are well known as a biological detergent of building material, which may serve as a permanent source of underground air pollutants [202].

Bioaerosol concentrations have strong time dependence and condition dependence. On the one hand, bioaerosol emissions during peak hours are much higher than those during off-peak hours [190,192,193,204,208], and concentrations in spring are higher than those in winter, which may correlate to higher outside temperatures and snow melting [196,202]. Meanwhile, the number of passengers and frequencies of trains may increase concentrations and transmissions of subway microbes [191,192,194,202,206,209,210]. Additionally, Heo and Lee suggested that concentrations of bacterial aerosols were affected by the number of passengers, but fungal aerosols were slightly affected by seasonal changes and human activities [211]. On the other hand, conditions of stations, tunnels, and facilities basically affect microbial concentrations. Depths of the subway stations positively correlate with microbial concentrations. Older subways presented worse microbial environments. Furthermore, as a major environmental control mean, polluted ventilation systems do great harm to microbial environments [190,196,210,212,213,214]. 

Pathogenic mechanisms of microbes are similar even in different environments. Crowded passengers increase microbial sources and deteriorate physical environments in subways. Increases in indoor temperature, humidity, and carbon dioxide indirectly promote microbial growth. Besides, crowded environments enhance antibiotic resistance and pathogen transmissions [191,197]. The pathogenicity potential of subway microbes has been controversial. Some research shows that fungal concentrations are significantly lower than bacterial ones in subways, and not severe enough to cause disease [202,206,215,216]. However, bacteria, together with fungal propagules, can cause respiratory disease. Risks of ‘‘mold’’-allergic diseases do exist during peak hours [202]. 

#### 4.2.4. Other Air Components

Other air compositions in subways also exert potential risks on passengers. CO, CO_2_, NO_2_, and other air compositions rapidly accumulate in subway stations and carriages due to confined spaces, huge populations, and various sources. CO usually remains at a relatively low level because fossil fuels and smoking are prohibited [81], but CO can affect human neurobehavior through combining with toluene and monocyclic aromatic hydrocarbons [171]. When it comes to CO_2_, its concentrations closely correlate with passenger numbers. When CO_2_ concentration exceeds 1000 ppm, occupants may complain about headaches, nose and throat ailments, tiredness, lack of concentration, and fatigue [81,217,218]. Additionally, radon exposes respiratory tissues to alpha radiation and increases incidences of lung cancer, but concentrations of airborne radon in subways are usually below admissible dosages [219,220,221,222].

#### 4.2.5. Mitigation Measures

In short, mitigation measures for IAQ aim at reducing pollutant concentrations and can be summarized into three aspects: (1) preventing external infiltration, (2) limiting internal sources, and (3) eliminating or diluting pollutants.

Firstly, air exchanges with ambient atmosphere may bring harmful substances into subways such as PMs, VOCs, microbes, allergens, moisture, and waste heat. Hence, aboveground ventilation shafts and entrances should be designed away from pollution sources. Furthermore, efficient filtrations are essential to prevent pollutant infiltrations. Filtration systems affect not only fresh air quality but also heat exchange rates of subway ventilation [223]. On the one hand, magnetic mesh filters have a certain capture efficiency for metallic PMs [224,225,226]. For easy cleaning and controllable magnetic field density, it is recommended to use electromagnets in a magnetic hybrid filter system [225,227]. On the other hand, composite filtration systems are not a simple superposition of purification technologies, but a combination of different technologies to achieve complementary advantages [228]. In recent years, HVAC systems for new subways are equipped with electrostatic purification devices in air-conditioning units or windpipes, which can effectively reduce PM concentrations and remove organic pollutants, bacteria, and microbes through physical separations [229,230,231]. Apart from magnetic filters and electrostatic purification devices, there are many applicable purification technologies to specific pollutants: (1) fiber filtration to PMs, microbes, and radon, (2) activated carbon to PMs, NO_2_, and VOCs, (3) photocatalysts to VOCs and microbes, (4) negative ion to PMs, bacteria, and fungi, (5) ultraviolet to bacteria and fungi, and (6) plasma to PMs, VOCs, bacteria, and viruses [228,231,232]. However, these air purification technologies may cause other pollution problems such as ozone, nitrogen oxides, and other harmful gases produced in the process of electrostatic disinfection [229].

Secondly, various internal sources are more challenging in confined subways. Pollutants from passengers are difficult to control, and toilets, cleaning rooms, dustbins, and facilities are inevitable subway operations. Hence, mitigations mainly depend on controlling pollutant transmissions and cleaner materials. On the one hand, early ventilation designs depend on simple fresh air systems and winds driven by thermal pressure and train motion that bring polluted airflows from tunnels to whole stations. Hence, unreasonable ventilations become a source of exposure, which is discussed in detail in the next chapter. Zonal controls, air curtains, and PSDs have been installed to prevent polluted airflows from flowing into passenger zones. On the other hand, preventing emissions of harmful substances can radically reduce environmental risks. Applying liquid dust suppressants to new ballasts, prohibiting diesel-powered trains or facilities, building along a straight, horizontal trajectory, designing a variable longitudinal profile railway, and slowing trains at sharp curves and high gradients may minimize wears of rail, wheels, and brakes, and then reduce metallic PM emissions. Besides, developments of materials that emit fewer and less toxic particles are a positive way forward [5,82,233,234,235,236]. When it comes to VOCs, controlling ground fossil-fueled vehicles effectively decreases the VOC concentrations in subways [177], and restricted use and prompt cleaning of spilled oils, solvents, deodorants, and decoration materials can reduce indoor sources. For bioaerosols, reducing PMs can limit bioaerosol formations. Meanwhile, it is essential to inhibit the growth of microbes through regulating thermal and humid conditions, reducing porous materials, maintaining environmental hygiene, and installing sterilization facilities. 

Thirdly, although adsorption and sterilization devices can gradually eliminate some pollutants, they cannot meet the instantaneous huge demand in the subway which accommodates a large number of passengers. Hence, exhausting or diluting air pollutants through sufficient air exchanges and flexible ventilation strategies dominates IAQ controls of subways. Displacement ventilations are efficient air exchange means widely used to reduce indoor pollutants. They supply fresh air at very low-velocity levels at or near the floor level and then rise, driven by thermal stratifications, to bring pollutants to the surface, which allows pollutants to leave breathing areas and be removed more easily [25]. Meanwhile, good hygiene and regular maintenance are of prime importance in reducing adverse effects of ventilation systems [23,190,191,194,202,204,206,213,237]. Additionally, based on aerodynamic characteristics of different pollutants, CFD (computational fluid dynamics) simulations are of great significance for formulations of ventilation strategies.

## 5. Ventilation as a Source of Exposure

While optimizing the physical environment, a subway ventilation system also carries potential health risks that cannot be overlooked, such as transmission of air pollutants and generation of uncomfortable draft sensations. A subway is a whole network with its own climatic conditions, which significantly affect the ambient airflow and pollutant transmission. Air pollutants can originate from a myriad of sources, including the external atmosphere, underground tunnels, cleaning chemicals, and equipment, and may spread throughout subway stations. To adjust the complex wind environment, flexible design and efficient operation of comprehensive mechanical ventilation and environmental control systems are required. However, ventilation systems that are in continuous operation and under heavy loads are more prone to pollution by PMs and microbes. The hygiene of ventilation systems is always a problem. It has been found that indoor microbe pollution is closely associated with poor ventilation system hygiene. Several mitigation measures have been proposed, including regular cleaning of baffle plates and filters, the use of cooling systems, and, particularly, strictly controlling temperature and humidity in subway stations [78,238,239]. Moreover, extensive and instantaneous air exchange may result in the formation of high-speed airflow. If coupled with the train-induced wind, the airflows can cause uncomfortable draft sensations and even skin, respiratory, or cardiovascular conditions. Furthermore, noise inside a subway station is difficult to transmit outside to the external environment and produces worse and longer-term effects due to the closed, sound-proof underground space. While accounting for only a limited proportion of the total noise in a subway station, noise generated by the ventilation system is the most persistent and should be minimized. In the following section, ventilation is discussed as a source of exposure associated with health risks from wind environment and noise perspectives.

### 5.1. Wind Environment

Wind environments in subways are affected by mechanical ventilation, outside winds, buoyancy-driven airflows, train-induced winds (piston winds), or a combination of the above factors. The airflows that spread air pollutants from one space to another may cause IAQ problems in multiple indoor areas. Airflows from various pollutant sources inside and outside subways all bring air pollutants into subway stations. Airflows generated by passengers and trains cause resuspensions of settled pollutants [192]. These airflows all increase concentrations of air pollutants and affect pollutant discharge efficiencies. Wind environments also depend on building geometry, pollution sources, thermal/fluid boundary conditions, and ventilation designs like ventilation rate, location of supply outlets and return outlets, and diffuser characteristics [240]. 

Unlike aboveground office and residential buildings, subways only have several ventilation vents or station exits with limited sizes for air exchanges. High ventilation rates and frequent facility utilizations require ventilation systems to keep their cleanliness and efficiency to ensure abundant clean air entering subways. Furthermore, some recent energy-saving studies propose to utilize piston winds through train movement [241,242,243,244] and buoyancy-driven airflows through hot pressing [245], which make wind environments and pollutant transmissions more complicated. Spaces and airflows in subways are so complex that they cannot merely introduce fresh air to dilute and then exhaust air outside. According to pollutant sources, pressure differences, and cleaning standards in different subway areas, ventilation needs flexible designs, predictive analyses, and real-time adjustments.

Fire evacuation is another serious potential risk associated with subway ventilations. Ventilations and smoke exhausts should not only exhaust smoke efficiently but also ensure the separation of humans and smoke because fire smokes have high temperatures and immediate risks to humans. On the one hand, fire smokes include several toxic gases, especially CO. CO can quickly cause dyspnea and poisoning symptoms. A high-density smoke also reduces the visibility of evacuees [246,247,248]. Besides, hot smokes and fires may damage subway constructions [249]. On the other hand, initial airflows are generated by piston effects or pressure differences and affect later movements of fire smokes in an initial period [250]. For a reasonable and effective evacuation strategy, it is indispensable to study the background airflows in subway microenvironments [20,21]. The strategy "go up and take the nearest exit to the surface" may not be the best response, because fire smokes or toxic airborne substances tend to use the same routes as fleeing passengers [248]. Tsukahara et al. simulated a large-scale and multistory station that had a fire source on a middle floor and concluded that downward evacuations could be more effective than upward ones [246]. However, dynamic evacuation guidance is more applicable and safe according to smoke distributions and passenger positions [248,251].

#### 5.1.1. Complex Airflows

Firstly, “subway climatology” was defined by Pflitsch in the late 1990s, which referred to natural airflows caused by temperature differences between subways and outer atmospheres. Because temperatures inside subways are generally higher than those outside from the late summer to winter, penetrating cold airs and rising warm airs at subway openings to external atmospheres drive natural airflow in tunnels. Therefore, such climates significantly affect dispersions of airborne substances [19,252,253]. For CFD combined with pedestrian simulations with tracer gas experiments [254], dynamic evacuation systems based on subway climatology take into account locations of passengers, transmissions of air pollutants, and subway climate at that time. These systems can identify the most endangered areas and guide passengers via an adaptive route using audio and visual techniques [20,21,248]. 

Secondly, piston winds affect human health through IAQ, thermal comfort, and draft sensation. Piston winds are generated by vehicle motion and blow through whole stations. Piston winds force a large number of air pollutants or cold air into subway stations because their speeds significantly exceed the design values of ventilation systems [255]. Hence, piston winds seriously disorder operations of ventilation systems [80,256,257]. In 2013, Pan et al. systematically reviewed the piston effect in subway stations from the aspects of formation mechanisms, simulation analyses, environmental effects, and control measures [242]. Based on the review of Pan et al., the latest health-related studies are reviewed. On platforms, piston winds are generally weaker in the middle than at both ends [257], and higher PM concentrations usually occur at train entry points of platforms due to air turbulences caused by piston wind [80,91,96,258]. Hence, passengers should reduce time spent on platforms and wait for trains in the middle parts of platforms, which requires mitigations of space designs, and platform staff need frequent breaks and to regularly change working positions [257]. In tunnels, number, geometrical structure, linkage angle, and location of vent shafts and train frequencies directly affect the performance of piston winds [241,259,260,261,262,263]. Additionally, barriers placed at tunnel outlets [264] and partitioning blocks installed along the middle of the tunnels [265] can improve ventilation performance.

Thirdly, without mechanical ventilation and piston wind, stack effects dominate airflows to move towards stairwells, elevator shafts, and ventilation shafts, and may spread fire smokes throughout subway stations. Preliminary designs of subway spaces and environmental controls decide the performance of stack effects. Under the same condition, areas and locations of shafts usually predominate airflows, and areas are more dominant than locations [250]. Furthermore, replacing vertical shafts with tilted shafts can eliminate boundary layer separations to optimize smoke exhausts [266]. Pressure differences induced by stack effects are much larger than those caused by other driving forces like gas combustions or winds [250,267,268]. Hence, the pressure settings of ventilation systems need flexible adjustments between different areas.

#### 5.1.2. Mitigation Measures

Mitigations of wind environments in subways are comprehensive systems, which require coordinated operations of multiple systems. Firstly, zonal control is the most feasible for complex thermal and wind environments in subways. Especially at transfer stations or multilevel stations, zonal control can meet various ventilation requirements to maintain a consistent indoor environment. Secondly, using and limiting piston winds are difficult problems to maintain environmental health in subways. Thirdly, air supplies closely correlate with draft sensations and ventilation efficiencies, which are common problems in subways. Fourthly, mechanical smoke exhaust should ensure safe evacuations of passengers in emergency situations such as fire or a biological attack. Lastly, dynamic ventilation systems can adjust ventilation strategies to stable subway environments and energy conservations through computer technology and real-time monitoring. Corresponding mitigation measures of the above five aspects are analyzed in detail below.

Firstly, zonal environmental controls and personalized ventilation systems can limit the spread of air pollutants to a certain extent and maintain a unified comfort level. In general, there are three zones in subways: station hall, platform, and tunnel. Each area is composed of multiple functional areas or rooms. In terms of health risk, platforms almost isolate from the outside atmosphere and are the most complex subway zones, which are directly affected by polluted airs from station halls and tunnels. Platforms gather numerous passengers and intractable pollution sources like toilets, cleaning rooms, and machine rooms [148,215]. Increasing fresh air volumes and installing high-efficiency filters in ventilation systems can effectively reduce pollutant concentrations [148]. To be more specific, displacement ventilation seems to be the most promising for creating a better IAQ and an acceptable thermal comfort level indoors [25,269]. Displacement ventilation based on square column attachment has superior performance of ventilation efficiencies and thermal comforts compared to traditional mixing ventilations [270,271]. Meanwhile, considering air velocity, air temperature, age of air, and relative warmth index (RWI), Liu et al. assessed air distributions by adopting three different air supply schemes (mixing ventilation, stratified air ventilation, and air curtain ventilation) for a subway platform and demonstrated that air curtain ventilation presented an appropriate velocity and temperature distribution [22]. Hence, air curtains can be installed at the boundaries of different subway areas to control airflows. Additionally, tunnel ventilations are another influencing factor of platform IAQ and affect airflow directions between tunnels and platforms. Tunnel fans can introduce outside air or exhaust inside air under different conditions. Tunnel ventilation may reduce both mass concentrations and number concentrations of PMs on platforms by over 50%, even in the presence of full-length PSDs [272,273]. Furthermore, although installing PSDs reduce PM concentrations in platforms, it increases PM concentrations in tunnels and still affects passengers’ health in trains [274].

Secondly, air curtains and PSDs are major devices to control piston winds. On the one hand, air curtains can effectively control unsteady airflows to improve ventilation efficiencies, ensure comfortable draft sensations, and reduce energy consumptions [243,255,275]. However, when the outside temperatures are below −10 °C in severe cold regions, warm-air curtains at the entrance individually consume massive energy and may not prevent intrusions of cold air from outside. In this situation, controlling speeds of trains entering and leaving stations can reduce cold air entering stations [276]. Meanwhile, changing air resistance coefficients and structures of traditional curtains may adjust relationships of inlet and outlet air volumes and make use of waste heat from subway operations [243]. On the other hand, PSDs prevent air pollutants, especially PM10, from tunnels to platforms. PSDs have five research areas including IAQ, fire smoke, thermal environment, energy conservation, and airflow analysis [219,277,278,279,280,281,282,283,284,285]. PSDs with controllable vents are wildly attentional environmental control systems. Through coordination between controllable vents and HVAC devices, these systems adjust operation modes based on platform heating loads, outdoor weather conditions, and tunnel air temperatures. With controllable vents closed in air-conditioning seasons, environmental control systems operate as traditional PSD systems to prevent polluted air from entering platforms. With controllable vents open in non-air-conditioning seasons, these systems operate as platform bailout door (PBD) systems and take full use of piston effects to ventilate and remove wasted heat from stations [244,283]. Meanwhile, PSDs prevent objects or passengers from falling off platforms and reduce noises from trains and fans [286].

Thirdly, velocities, temperatures, and locations of air supplies directly affect environmental health and human health. The vertical temperature gradient presents a significant variation in subway stations [22]. Deep and multi-story stations are a trend of subway designs, which exacerbate energy losses of downward airflows and cause poor ventilation areas [247]. A reasonable design of air supply systems can provide adequate fresh air and comfortable draft sensation in every subway area and make the ‘dead zone’ of ventilation disappear [287]. Meanwhile, air supplies should maintain consistent draft and thermal sensations when passengers move from one area to another [35]. Additionally, Wang et al. studied coupling airflows between air-conditioning air supplies and piston winds in a platform without PSDs. They showed that piston winds led to an increase in heat dissipation from human skin. Comfort levels under air-conditioning airflows alone were better than those of coupling airflows. However, these coupling airflows may improve transitional thermal comforts and have huge energy-saving potentials in air-conditioning systems by increasing air temperatures of air supplies [288,289]. 

Fourthly, mechanical exhaust systems can ensure passengers’ safety in case of fires or biochemical attacks. On the one hand, due to the flexibility and reliability, hybrid exhaust systems are more suitable for subway environments. Integrated utilization of mechanical and natural ventilation effectively inhibit fire smoke dispersions and decrease toxic substance concentrations [290]. Smoke exhaust systems in tunnels and platforms can operate collaboratively to deal with different situations [291]. Meanwhile, smoke control systems in subways consist of tunnel ventilation fans, under platform exhaust systems, smoke evacuating gates, and platform edge doors. Smoke control systems actively control smoke movements and ensure passenger safety [250,267]. On the other hand, there are some measures to improve efficiencies of existing smoke exhaust systems, such as breadthways ventilations in low-height platforms without a ceiling duct [287], equivalent vent velocities in long smoke removal pipes [292], heat exhaust coefficients for tunnel transversal smoke extraction system [293], and ventilation strategies for smoke-free staircases [294].

Lastly, subway IAQs significantly change in real-time due to various time-dependent factors such as subway schedules, passenger loads, and outdoor climates. [295]. For quick responses in controlling subway IAQs, real-time monitors, multi-objective optimizations, and dynamic control systems can improve the performance of ventilation systems by adjusting equipment, strategies, and rates [273,295,296,297,298,299,300,301,302]. Variable air volume systems can dynamically meet ventilation requirements with less energy consumption and comfortable thermal condition [303,304]. Marzouk and Abdelaty presented an application that utilized a wireless sensor network and building information modeling to monitor thermal conditions in subways [305]. Furthermore, multivariate monitoring and local interpretation of IAQs better isolate air quality characteristics that vary seasonally and allow for more specific monitoring of air pollutants [306]. As discussed above, outdoor air quality (OAQ) around subways significantly affects the IAQ. Hence, OAQ monitoring systems with feedback and feedforward controllers can forecast IAQ changes and adjust ventilation strategies in advance [299]. Additionally, to reduce errors caused by sensor faults, an air pollutant prediction model based on an adaptive network-based fuzzy inference system was used to detect sensor fault, and a structured residual approach with maximum sensitivity method was used to identify and reconstruct sensor faults existing in subway system [307]. However, dynamic data of the above studies were from periodic measurements of air quality monitoring stations, which may be insufficient to provide precise data for assessing individual environmental risks. Wearable sensors are more suitable to provide estimations of IAQ in the proximity of passengers, which can provide necessary information on health risks for passengers to make travel arrangements that minimize exposure to polluted air [308].

### 5.2. Noise

Noise generated by ventilation systems constitutes a limited part of subway noise but lasts the longest. Long-term exposure to environmental noise can have a number of health effects [309,310]. The primary sources of subway noise include subway operation, public broadcasting, and human activities. Regular noise caused by subway operation can be categorized into several types, namely, wheel/rail noise, machine noise, traction noise, and brake noise, of which, wheel/rail noise contributes the most to the total noise. A questionnaire-based study conducted by Wang et al. found that over 90% of the respondents agreed that subway stations, particularly transfer stations and during rush hours, were noisy [311,312]. Therefore, reducing the noise level is beneficial to ensuring a healthy subway environment. There are some conflicts between sound insulation and ventilation requirements. For example, PSDs can effectively reduce train and fan noise in tunnels [311] but affect the use of piston winds. In addition, tubular subway spaces are usually long, narrow, and low, and therefore have long reverberation times and poor language clarity [312]. Spatial acoustic designs for conventional subways have a multitude of defects and require further research.

#### 5.2.1. Continuous Noise

Noises have adverse effects on psychological, biological, immunological, and endocrine systems [313]. Because few studies focus on the proportion and quantity of ventilation noise, this paper summarizes related effects based on whole noises in subways, which include auditory, extra-auditory, and mental effects. Firstly, there are some long-term and incremental auditory effects such as noise-induced hearing loss, tinnitus, and unnoticeable diminution in hearing acuity. Auditory effects are caused by mechanical damage to inner ears. Subway noises exceed limit values recommended by Environmental Protection Agency and WHO, which indicate exposure duration should be shorter than 45 minutes under a mean noise level of about 85 dBA. The maximum noise levels of platforms or carriages were even higher than 100 dBA [314]. Secondly, extra-auditory effects include hypertension, disturbance in hormonal secretion, cardiovascular diseases, and obesity. Exposure to high levels of noise may result in elevation of cholesterol, triglycerides, lipoproteins, and blood pressure and then affect cardiovascular, respiratory, and central nervous systems [313,314]. Thirdly, mental effects include psychological pressure, annoyance, frustration, fatigue, sleepiness, apathy, insomnia, and memory problems. Although noise-related effects are explained in relation to central nervous systems, secondary and tertiary reactions are not controlled by brain cortexes, which may reduce corporal and mental functions in the long-term. Exposure to noise in the long-term also causes a reaction of exhaustion or defeat which leads to disturbance in the secretion of some hormones such as the growth hormone, catecholamine [315,316], possibly followed by dysfunction of immune systems. Additionally, noises have adverse effects on concentration, work capacities, human communications, and increase accident risks [314,317].

#### 5.2.2. Mitigation Measures

Reduction measures for subway noise can be classified into four categories: source mitigations of tracks, such as braking mechanisms, ventilation machines [314], sound insulation devices like acoustic enclosures [311] or PSDs with microperforated panels [311,312], personal hearing protection devices [314], and acoustic designs for subway spaces [312]. Additionally, temperature and humidity in subways can affect values of sound reverberations and speech intelligibility [318]. Health risk assessments of ventilation noises need further studies to provide exposure thresholds and environmental indices.

## 6. Discussion

Subways have gradually become the most used UUS for urban residents on a daily basis. Therefore, it is important to pay attention to exposure risks associated with subway microenvironments and establish their control standards, evaluation systems, and mitigation toolbox [319]. However, there is insufficient awareness of the potential for long-term health effects of the subway environment. Subway passengers need a healthy environment with comfortable temperature, humidity, and draft conditions, as well as clean indoor air. Ventilation is the most important measure for adjusting the physical environment in a subway station but may also cause serious health issues in the absence of rational design and continuous operation.

### 6.1. Dual Effect of Subway Ventilation on Environmental Health

Through a narrative literature review, a distinguishing feature of subway ventilation was uncovered in this study, that is, subway ventilation exerts a dual effect, as both a mitigation measure and a source of exposure. The accumulation of heat, moisture, and air pollutants inside a subway station dictates the health level of its environment in normal time. Deterioration of the thermal environment can cause adverse responses in almost every body system, and then increase disease incidence. Temperature and humidity also exert indirect health effects by affecting other environmental factors such as PAQ, microbial pollution, and pressure difference. When it comes to IAQ in subways, PMs, VOCs, and bioaerosols are sources of exposure of the most concern. Metallic PM and the resulting oxidation potentials are viewed as representative risks that affect environmental health in subways and have been extensively analyzed through pathological studies and simulations. Health risks of VOCs, which are carcinogenic and genetically toxic, may be underestimated due to their complex sources, delayed effects, and insufficient toxicity studies. Humid environments and ventilation systems provide conditions for the growth and spread of microbes and bioaerosols. These problems can be addressed using efficient ventilation. However, ventilation is a double-edged sword, due to the lack of a source of totally clean air in subways. First, natural airflows caused by pressure difference and piston wind may bring polluted air or fire smoke throughout the subway station and thereby worsen IAQ. Then, the humid condition, huge load, continuous operation, and fixed installation all render it difficult to maintain a clean ventilation system. Moreover, high-speed air supply causes uncomfortable draft sensations that may affect passengers’ integumentary, respiratory, and cardiovascular systems. Finally, noise will be amplified in the closed subway space. Continuous noise generated by ventilation systems should be minimized. Overall, ventilation exerts not only direct effects on human health and comfort but also indirect effects by affecting other environmental factors. Notwithstanding, ventilation is an indispensable measure for controlling the indoor environment in a subway station. Hence, it requires a more comprehensive design, flexible adjustment, and continuous maintenance.

### 6.2. Reliable and Specific Environmental Health Risk Assessment

Further research is required to study some problems related to environmental health in subways. In terms of medicine and epidemiology, the exposure threshold, disability-adjusted life year (DALY), acceptable daily intake (ADI), and other indices are controversial on some risks. The correlations and differences between experiments in vitro and in vivo complicate the formulation of these indices. It is, therefore, necessary to take into consideration whether there are environmental influences or interactions between pathogenic factors when studying pathogenic mechanisms and exposure thresholds. Long-term epidemiological surveys may be more reliable than toxicity tests. Furthermore, life cycle impact assessment models require further improvement to comprehensively assess the health effects and not only use physicochemical and toxicological properties but also other relevant parameters combined with environmental characteristics, such as the USEtox method [320]. More specifically, the potential effects of indoor humidity on air pollution and human health can still benefit from further research. More research into subway air pollution should be conducted to examine the suitability and reliability of the reference aboveground indices. Additionally, more research is also required to optimize detection technology and exposure assessment techniques to reduce equipment-related differences between studies. 

Based on reliable exposure thresholds and maximum acceptable concentrations, environmental health indices can be used to control health risks more effectively. A number of countries and organizations have published health-based environmental control guidelines or standards, as shown in Table 5. In addition to these intuitive indices, attention should also be drawn to some potential microenvironmental changes. For example, sudden temperature changes can severely affect comfort and even trigger acute cardiovascular events when passengers pass through different areas inside or outside a subway station. This is a common problem in most subway stations, particularly those that serve as major transport hubs and those in cold or tropical climates. This problem may be addressed by dynamic thermal comfort assessments and body condition monitoring. Through zonal ventilation control, the gradual change or stable condition of the thermal environment in a subway station can be adjusted on the temperature difference and human comfort. Additionally, it is also necessary to establish assessment systems specifically for subway IAQ that fully account for the pathogenic and spatiotemporal characteristics of pollutants in subway environments.

### 6.3. Ventilation Mitigation to Improve Environmental Health

Ventilation not only significantly affects the thermal and humid environment in a subway station but also plays a vital role in air pollutant transmission and air pollutant-related infection. On the one hand, integrated and intelligent ventilation systems can improve IAQ and conserve energy in subway stations [80,233,241,242,243,244]. In a subway station, piston winds, hot pressures, the stack effect, and the subway climate can all increase the ambient airflow, regardless of whether the mechanical ventilation system is in operation. However, the airflow may originate from sources of pollution inside and outside the subway station. Hence, it is essential to install composite filtration systems to purify air. Some filtration-related problems need, however, to be tackled, including rapid efficiency reduction, difficulties in removing built-up pollutants, emergency disinfection, sterilization capacity, and automated monitoring, cleaning, and alarm technologies. On the other hand, zonal and dynamic control systems can be employed to control the complex and changeable wind environment in a subway station and adjust the mechanical ventilation mode in a flexible and timely manner to prevent the spread of air pollutants or fire smoke. When it comes to specific measures, air curtains, PSDs, and interior space design are effective measures to block polluted airflow and fire smoke. PSDs with controllable vents can be used to simultaneously take advantage of piston winds and block PMs by installing nanofiber filters [321]. However, although the above measures can block complex airflow movement, ventilation strategies for piston wind should be adjusted flexibly in different climate zones. For example, subway stations in cold zones are shielded by PSDs and air curtains to reduce the penetration of external cold air caused by piston winds in winter. But these stations in tropical zones need piston winds in the summer to release internal heat and reduce HVAC loads. Therefore, how to design and control ventilation shortcuts for piston winds so that pollutants and heat can be exhausted quickly from platforms to outside might make sense. Additionally, an understanding of the aerodynamic and reproductive characteristics of air pollutants can help formulate corresponding environmental indices and ventilation strategies.

**Figure 3:**
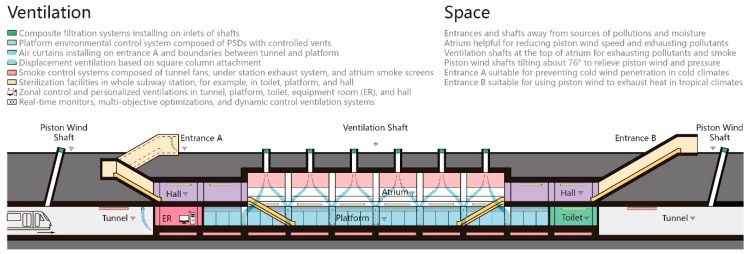
An ideal ventilation and space environment for subway stations.

Another potential problem is various interactions between different ventilation strategies. On the one hand, ventilation measures described in the previous paragraph inevitably result in an inconsistent environment in a subway station characterized by variable conditions (temperature, humidity, air quality, pressure, wind speed, and age of air) as well as dirty “dead zone” and uncomfortable draft sensations. Therefore, subway ventilations need comprehensive design and flexible adjustment through real-time monitoring, demand forecasting, timely feedback, and other dynamic ventilation strategies. On the other hand, synergistic or inhibitory interactions exist in different ventilation strategies, which echoes the dual effect of subway ventilation. Large air volume can effectively discharge air pollutants and improve IAQ, but also reduce efficiencies of cooling or heating systems, affecting the thermal comfort of passengers. Meanwhile, the ventilation strategy of using piston wind to exhaust heat can cause draft sensations, especially in narrow spaces, and cause the spread of air pollutants to whole stations. Among these interactions, a significant problem is some defects of ventilation paths and space design in traditional subway stations. Therefore, it is necessary to emphasize the impact of space design on environmental health in subway stations. Arguably, a rational space design is the basis for efficient ventilation. Adverse effects of unreasonable subway spaces are hardly compensated by traditional ventilation-based design patterns of environmental control systems. Compared with ventilation systems, spatial connection and airflow movement dominate the spreading of airflow movement and thermal comfort in subway stations, which is a basic problem more or less neglected by previous studies and engineering design. Meanwhile, spatial acoustic design, to a certain extent, affects subway noise control. The subway environment is significantly affected by its interior space factors, including shape, size, material, connection and separation of spaces, and the number, location, and size of vertical shafts, stairs, and exits. In architectural and interior design, there are often some spaces or components that block smoke movement and exhaustion [250]. For example, vertical walls will result in smoke backflow and increase smoke temperature under the ceiling [249]. Compared to conventional tubular spaces, large atria are beneficial when it comes to ensuring fire safety in subway stations because they can allow smoke and heat to accumulate close to the ceiling and accelerate smoke exhaustion [290,322]. In a subway tunnel, partition blocks installed between two tracks can reduce the airflow effect on one track induced by train motions on the other track and enhance the piston effect [265]. With the integrated utilization of underground spaces and developments of underground engineering, further research is required to investigate more systematic spatial patterns on the improvement of environmental health. For example, atriums between station halls and platforms provide significant relief from high-speed piston winds, because they eliminate narrow airflow lanes, like stairs channels. Meanwhile, atriums, combined with effective smoke blocking devices like smokescreen and smoke-preventing air curtains, can effectively transport air pollutants and fire smoke to the top of atriums and then exhaust outside through ventilation shafts. Compared with the conventional strategy of limiting smoke in horizontal layers, this strategy has obvious advantages in exhausting smoke, reducing thermal damage of constructions, and organizing evacuation paths. Furthermore, atriums are conducive to shaping a comfortable spatial scale that may reduce the psychological effects of underground spaces. Based on the discussion in this subsection, a prototype of subway stations with ideal ventilation and space environments, which is derived from various research findings and integrated into a conceptual diagram, is shown in Figure 3.

## 7. Conclusions

This study clarifies the complex relationship between ventilation and human health in subway stations from the perspectives of pathology, epidemiology, engineering, and built environment. Recommend exposure thresholds or values from medical and epidemiological studies that offer a basis for health risk assessment and environmental control are discussed. Health risk assessments of subways should further integrate specific toxicology research of subway pollutants, long-term epidemiological data, and environmental characteristics of subways. Based on these data, engineering measures can be implemented to effectively improve environmental health in subway stations. In short, ventilation exerts a notable dual effect on environmental health in subway stations. Ventilation, on the one hand, is a dispensable measure for optimizing the physical environment in a subway station and, on the other hand, can potentially negatively affect its wind and acoustic environment. Wind environments are more complex in subway stations than in aboveground buildings due to various pollution sources, limited ventilation inlets/outlets, and strong background airflows caused by piston wind and subway climate. Ventilation systems in subway stations not only should introduce clean, fresh air and dilute and/or discharge air pollutants, but also should be controlled zonally and adjusted in real-time. It is necessary to simulate and analyze the effects of variable airflow combined with the aerodynamic behavior of various air pollutants. Additionally, space optimization, as the basis of efficient ventilation, has not received enough attention in the past due to limited construction technologies and immature professional cooperation. Unreasonable space connections and ventilation paths may cause a series of adverse effects and contradictions, which are hardly compensated by ventilation systems and devices. In the traditional design pattern of a subway environment, space precedes ventilation, and ventilation remedies space, which should be replaced by a more comprehensive design pattern. The coupling relationship between space and ventilation should be considered at an early stage, and the organization strategy of internal and external airflow should be determined according to regional climate and environmental conditions.

## Figures and Tables

**Table 1:** Thermal environment.

Risk Factors	Environmental and Health Effects	Mitigation Measures
High temperature	Environmental effects: excessive heat Health effects: heat stroke, heat syncope, heat cramp, dehydration, hypertension, organ dysfunction, anxiety, dysphoria	Improving efficiencies and designs of heating, ventilating, air conditioning, and filtration systems Rational designs of entrances and shafts to prevent outdoor rain, moisture, and cold air entering subway stations Reducing internal water sources like underground water seepage, toilets, cleaning rooms, and equipment Temperature and humidity independent control system Calculation and prediction of heat and humidity loads Identifying reliable exposure threshold, environmental indices, and comfort evaluation (three other environmental factors also face this challenge: environmental standards specific to subways based on more research on toxicities of subway risks and their long-term effects.)
Low temperature	Environmental effects: cold-heat change Health effects: atherosclerosis, myocardial infarction, ischemic heart disease, dehydration, hypertension, vasoconstriction in respiratory tract, rhinitis, obstructive airway, susceptibility to infection	
High humidity	Environmental effects: microbial growth and air pollution Health effects: perceived air quality (PAQ), skin and airway symptoms, functional attenuations of reproductive, muscle, and skeletal systems	
Low humidity	Environmental effects: virus survival Health effects: PAQ, inflammatory reactions in nasal and eyes, sensory irritations, work performance	

Review and analysis according to the literature [14,15,22,32,33,34,35,36,37,38,39,40,41,42,43,44,45,46,47,48,49,50,51,52,53,54,55,56,57,58,59,60,61,62,63,64,65,66,67,68,69,70,71,72,73,74,75,76,77,78].

**Table 2:** Indoor air quality.

Risk Factors	Environmental and Health Effects	Mitigation Measures
Particulate matter	Environmental effects: transmission of infectious diseases Health effects: cellular inflammation and oxidation reaction in multiple body systems, heart disease, stroke, asthma, allergy, cancer, genotoxicity	Preventing external infiltration: Aboveground shafts and entrances away from pollution sourcesInstalling and cleaning composite filtration systems Limiting internal sources: Maintaining indoor hygiene especially in cleaning rooms, dustbins Reducing pollutant emissions from fuel, wheel, brake, oil, solvent, deodorant, and decoration material Installing air curtain, platform screen door (PSD), and other devices Eliminating or diluting pollutants: Sufficient air exchange and flexible ventilation strategy Optimizing ventilation mean and equipment Good hygiene and regular maintenance of ventilation system Installing adsorption and sterilization devices
Volatile organic compound	Health effects: PAQ; asthma, allergy, nasal irritation; eye irritation; cancer; genotoxicity; neurological symptom	
Bioaerosol	Environmental effects: propagation and transmission of microbes, antibiotic resistance and transmission efficiency of pathogens Health effects: pathogenic mechanisms are similar in different environments	
Others	Environmental effect: interactions with other air pollutants Health effects: CO: neurobehavioral effect CO_2_: headache, nose and throat ailments, tiredness, fatigue Radon: alpha radiation, lung cancer	

Review and analysis according to the literature [5,8,9,10,11,12,14,15,16,17,18,26,27,28,29,30,31,79,80,81,82,83,84,85,86,87,88,89,90,91,92,93,94,95,96,97,98,99,100,101,102,103,104,105,106,107,108,109,110,111,112,113,114,115,116,117,118,119,120,121,122,123,124,125,126,127,128,129,130,131,132,133,134,135,136,137,138,139,140,141,142,143,144,145,146,147,148,149,150,151,152,153,154,155,156,157,158,159,160,161,162,163,164,165,166,167,168,169,170,171,172,173,174,175,176,177,178,179,180,181,182,183,184,185,186,187,188,189,190,191,192,193,194,195,196,197,198,199,200,201,202,203,204,205,206,207,208,209,210,211,212,213,214,215,216,217,218,219,220,221,222,223,224,225,226,227,228,229,230,231,232,233,234,235,236,237].

**Table 3:** Wind environment.

Risk Factors	Environmental and Health Effects	Mitigation Measures
Complex airflows	Environmental effects: Spreads of air pollutant and fire smoke Overlay path of evacuee and fire smoke Inconsistent thermal and wind environments in different zones Reducing ventilation efficiency and heat exchange rate Accumulations of heat, moisture, and pollutants Strong draft sensation and unstable thermal sensation Health effects: Aggravation on other environmental factors Acute and serious risks during emergencies like fire and terrorist attack Cardiovascular, respiratory, skin, nervous, and auditory responses	Zonal environmental control, personalized ventilation, and variable air volume system Limiting piston wind or other airflows through PSD and air curtain Using piston wind through PSD equipped with controllable vent and (nanofiber) filter Hybrid exhaust system and dynamic evacuation system Increasing fresh air volume and installing high-efficiency filter Optimizing velocity, temperature, and location of air supply Dynamic ventilation system equipped with real-time monitor, multivariate monitoring, multi-objective optimization, feedback and feedforward controllers, or wearable sensor

Review and analysis according to the literature [18,19,20,21,22,23,24,238,239,240,241,242,243,244,245,246,247,248,249,250,251,252,253,254,255,256,257,258,259,260,261,262,263,264,265,266,267,268,269,270,271,272,273,274,275,276,277,278,279,280,281,282,283,284,285,286,287,288,289,290,291,292,293,294,295,296,297,298,299,300,301,302,303,304,305,306,307,308].

**Table 4:** Noise.

Risk Factors	Environmental and Health Effects	Mitigation Measures
Continuous noise	Health effects: Auditory effects: noise-induced hearing loss, hearing acuity, tinnitus Extra-auditory effects: hypertension, disturbance in hormonal secretion, obesity, cardiovascular disease Psychological effects: pressure, annoyance, frustration, fatigue, sleepy, apathy, insomnia, cognitive impairment	Source mitigations of track, brake, ventilation machine Sound insulation devices like acoustic enclosures and PSD with microperforated panel Personal hearing protection device Acoustic design for subway space

Review and analysis according to the literature [17,309,310,311,312,313,314,315,316,317,318].

**Table 5:** Environmental control indices of relevant standards or guidelines.

Environmental Control Factors Related to Ventilation	WHO Guideline for Indoor Air Quality and Occupational Health (WHO)	The WELL Building Standard-V1 (US Green Building Council, ASHRAE)	Hygienic Indicators and Limits for Public Places (China)	Indoor Environmental Input Parameters (CEN)	Environmental Health Management Standard for Buildings (Japan)
TEMP (°C)	16~28 (Healthy residence)	Graphic Comfort Zone Method	26~28	20~26	17~28
RH (%)	45~75 (Microbial inhibition)	30~50	40~65	30~65	40~70
PM_2.5_ (μg/m³, 24-hour mean)	25	35	-	25	150 (Suspended dust)
PM_10_ (μg/m³, 24-hour mean)	50	50	150	-	
HCHO (μg/m³, 1-hour mean)	100	27 ppb	100	100	100
TVOC (μg/m³, 8-hour mean)	300	500	600	300~1000	400
Total bacteria (CFU/m³)	200~500	-	4000	-	-
CO (mg/m³, 8-hour mean)	10	10	10	15	10 ppm
CO_2_	1000 ppm	800 ppm	0.15%	1000~2000 ppm	1000 ppm
O_3_ (μg/m³, 8-hour mean)	100	100	160	60	0.1 ppm
Radon (Bq/m³, annual mean)	100	148	400	100	-
Air change flow (m³/(h·pers))	-	41.65	30	25.2	-
Wind speed (m/s)	-	0.15	0.5	-	0.5
Noise (dB(A))	85	40	85	60	60

Abbreviations: WHO (World Health Organization); CEN (Comité Européen de Normalisation, European Committee for Standardization); ASHRAE (American Society of Heating, Refrigerating and Air-Conditioning Engineers); TEMP (Temperature); RH (Relative Humidity); PM (Particulate Matter); TVOC (Total Volatile Organic Compound). “Indoor environmental input parameters” refers to the recommended parameters of Category Ⅱ for normal level in the standard “Indoor environmental input parameters for design and assessment of energy performance of buildings addressing indoor air quality, thermal environment, lighting, and acoustics” published by CEN.
